# Pin1 aggravates renal injury induced by ischemia and reperfusion in rats via Nrf2/HO-1 mediated endoplasmic reticulum stress

**DOI:** 10.1590/acb370101

**Published:** 2022-04-08

**Authors:** Honglin Yu, Guanjun Jiang, Wei Hu, Changgeng Xu

**Affiliations:** 1 MD. Department of Radiology – The First Affiliated Hospital of Anhui Medical University – Anhui, China.; 2 MD. Department of Urology – The Fifth Hospital of Wuhan – Wuhan, China.; 3 MD. Department of Andrology – The First Affiliated Hospital – Hengyang Medical School – University of South China – Hunan, China.; 4 MD. Department of Urology – The Central Hospital of Wuhan – Tongji Medical College – Huazhong University of Science and Technology – Hubei, China.

**Keywords:** NIMA-Interacting Peptidylprolyl Isomerase, Ischemia, Reperfusion, NF-E2-Related Factor 2, Heme Oxygenase-1, Endoplasmic Reticulum Stress

## Abstract

**Purpose:**

To investigate the role of peptidyl-prolyl cis/trans isomerase 1 (Pin1) on renal ischemia-reperfusion (I/R) injury and underlying mechanism.

**Methods:**

By establishing the in vitro and in vivo models of renal I/R, the role of Pin1 was explored by using molecular assays.

**Results:**

In renal I/R, endogenous Pin1 level was up-regulated in I/R-impaired kidney. Suppression of Pin1 with juglone afforded protection against I/R-mediated kidney dysfunction, and reduced I/R-induced endoplasmic reticulum (ER) stress in vivo. Consistent with the in vivo results, repression of Pin1 with juglone or gene knockdown with si-Pin1 conferred cytoprotection and restricted hypoxia/reoxygenation (H/R)-driven ER stress in HK-2 cells. Simultaneously, further study uncovered that Nrf-2/HO-1 signals was the association between Pin1 and ER stress in response to renal I/R. In addition, Nrf-2/HO-1 signal pathway was inactivated after kidney exposed to I/R, as indicated by the down-regulation of Nrf-2/HO-1 levels. Furthermore, inhibition of Pin1 remarkably rescued the inactivation ofNrf-2/HO-1.

**Conclusions:**

Pin1 modulated I/R-mediated kidney injury in ER stress manner dependent on Nrf2-HO-1 pathway in I/R injury.

## Introduction

Renal ischemia-reperfusion (I/R) injury, considered as one of the major stimuluses that put kidney in susceptibility to acute kidney injury (AKI), might impair kidney function after kidney transplantation and partial nephrectomy^1^. Notably, the mechanisms whereby I/R results in kidney injury are complicated and multiple, with various injured stimuli implicated in the pathogenesis, including apoptosis, ER stress, autophagy, and oxidative stress^2^^,^ 3. Previous studies have been committed to explore promising therapeutics for protecting against I/R-induced AKI, such as remote ischemic preconditioning, postconditioning and new pharmacological interventions^4^^,^ 5. However, little work has been validated in preclinical phase. Therefore, it is necessary to determine the potential mechanisms and investigate new therapeutic targets in the field of renal I/R.

Pin1 confers its function by efficiently promoting the conformational alterations of the substrate proteins through specifically identifying phosphorylated Ser/Thr-Pro peptide bonds^6^. The Pin1 catalyzes isomerization of the substrate proteins and alters the functional activities of targeted phosphoproteins, to control many biological processes, including tumorigenesis and development, gene transcription, and redox balance^7^. A recent report indicated that Pin1 was related to the progression of ischemic stroke by targeting p53 transactivation^8^. Another study indicated that suppression of Pin1 restricted intestinal ischemic injury and these effects mediated by the p66Shc-induced mitochondrial homeostasis^6^. However, whether Pin1 mediates kidney dysfunction in the context of I/R injury remains to bring to light.

Nuclear factor-erythroid 2-related factor 2/heme oxygenase-1 (Nrf2/HO-1) signal pathway is associated with various cell biological processes, including ER stress. Nrf2/HO-1 was reported to be the upstream regulator of ER stress during AKI^9^^,^ 10. However, whether Pin1 is correlated with Nrf2/HO-1 expression and ER stress still remains unknown. Herein, we showed that suppression of Pin1 extenuated I/R-induced ER stress in rats and explored the involved mechanisms.

## Methods

### Animal, I/R model establishment

Male Sprague-Dawley (SD) rats (180–200 g, 8 weeks old) were obtained from Shanghai Science Academy Animal Center (Shanghai, China). The project was conducted in line with the Principles of Laboratory Animal Care (NIH Publications No. 8023, revised 1978). All animal procedures were approved by the Institutional Animal Care and Use Committee of Anhui Medical University (Approval number: LLSC20210858). Animals were kept in a food and water freely available environment with a 12-h light and 12-h dark cycle. The I/R model was performed according to the well-established method^11^. Briefly, a midline abdominal incision was made and both kidneys were exposed. The main renal arteries and veins were identified using a stereotactic microscope, followed by a right nephrectomy. Then the left renal arteries and veins were occluded for 45 min with nontraumatic microaneurysm clamps. After 45 min of renal ischemia, the abdomen was reopened and the clamps were removed. The kidneys were inspected for at least 1 min to ensure restoration of blood. The abdomen was closed with continuous 4-0 polypropylene sutures. Renal tissues and blood were obtained at various reperfusion time points (6, 12, 24 h). Sham surgery consisted of an identical procedure without application of the microaneurysm clamps on the left renal arteries and veins.

### Animal groups

Seventy-two rats were randomly divided into the following groups (n = 8): Sham group (n = 8)—rats were only exposed to Sham surgery; I/R 6 h group (n = 8), I/R 12 h group (n = 8), and I/R 24 h group (n = 8)—rats suffered from ischemia for 45 min and various reperfusion periods; I/R group (n = 8)—rats were subjected to I/R injury insult only; I/R+DMSO group (n = 8)—rats were injected the equal DMSO before I/R establishment; I/R+juglone (2.5 mg·kg^–1^) group (n = 8),I/R+juglone (5 mg·kg^–1^) group (n = 8), I/R+juglone (10 mg·kg^–1^) group (n = 8)—rats were intraperitoneally injected with various doses of juglone (once a day), three consecutive days before I/R surgical protocol.

### Serum assays

The serum concentrations of blood urea nitrogen (BUN) and Cr were assessed by the commercial kit (Nanjing Jiancheng company, China) based on the manufacturer’s instruction.

### Cell culture and treatment

The human kidney cell line (HK-2) was cultured in complete Dulbecco’s modified eagle medium (DMEM) (Invitrogen, USA) supplemented with 10% fetal bovine serum (FBS) and 1% penicillin and streptomycin in a humidified environment containing 5% CO_2_ at 37 °C. To established a mimic hypoxic atmosphere of H/R model, cells were incubated in a hypoxic incubator with 1% O_2_, 94% N_2_, and 5% CO_2_ for 12 h. Then to reoxygenation, the cells were cultured in a normoxic incubator for 2, 4 and 6 h. The control cells were cultured in the humidified atmosphere with 5% CO_2_ at 37 °C.

### Small interfering RNA (siRNA) transfection

For transfection, HK-2 cells were cultured in medium without FBS. Specific siRNAs or negative siRNAs (GenePharma, China) were transfected into cells using Lipofectamine 2000 reagent for 6 h. Then, the medium was replaced with complete medium supplemented with 10% FBS.

### Hematoxylin and eosin (H&E)

Kidney tissues were sectioned into 4 μm thick, followed by fixed, paraffin-embedded. Then, the sections were used to assess histologic damages by H&E. Histological evaluations were performed according to the previously established criteria by Jablonski *et al.* 12. Morphological alterations were measured by two pathologists who knew nothing about the treatment group.

### Reverse transcription polymerase chain reaction (RT-PCR)

Total RNA from cells or renal tissues were prepared using Trizol (Invitrogen, USA), followed by synthesis into cDNA using the cDNA Reverse Transcription Kit (Applied biosystems, USA). qPCR was carried out using the SYBR Green (Bio-Rad). Primer sequences targeting specific genes were listed as follows: R-Pin1: 5΄-GCTCAGGCCGTGTCTACTACTTC-3΄ (F), 5΄-TCCGAGATTGGCTGTGCTTC-3΄ (R); R-β-actin: 5΄-TGCTATGTTGCCCTAGACTTCG-3΄ (F), 5΄-GTTGGCATAGAGGTCTTTACGG-3΄ (R). Relative expression levels of mRNA were measured by Ct values and normalized to GAPDH.

### Western blotting

Total protein samples were prepared from cells or renal tissues using a radio immunoprecipitation assay (RIPA) protein extraction kit. Primary antibodies used in this section were listed as followed: Pin1 (1:1000, Abcam), p-eIF2α (1:1000, Cell Signaling Technology), eIF2α (1:1000, Cell Signaling Technology), CHOP (1:1000, Cell Signaling Technology), GRP78 (1:1000, Cell Signaling Technology), Nrf2 (1:1000, Abcam), HO-1 (1:1000, Cell Signaling Technology), and β-actin (1:5000, Boster Biological Technology). Each band of density was measured using Image J.

### Cell Counting Kit-8 (CCK-8)

Cell viability was measured with the CCK-8 detection kit (Beyotime Biotechnology, #C0037) according to the instructions.

### Statistical analyses

Data collected from each experimental group were presented as means ± standard error of mean (SEM). The normal distribution of the data was evaluated by Shapiro–Wilk test. Since data normality distribution was met, the groups were compared by an analysis of variance (ANOVA) followed by Tukey’s multiple comparison tests to determine which groups differed with pairwise comparison. The priori sample size and post-hoc power analyses were calculated using the G-power package. The sample size was calculated for ANOVA test, which was used to test the main hypothesis of the study. As a result of the sample size analysis performed using previous study knowledge, it was found that 80 rats, 8 in different groups, needed to be involved in the study to reveal the significant differences in the groups using 80% power (1-β = 0.80), α = 0.05 error (95% confidence interval) with a two-sided hypothesis. P < 0.05 was considered statistically significant.

## Results

### Pin1 level up-regulated in I/R injured kidney

**Figure 1:**
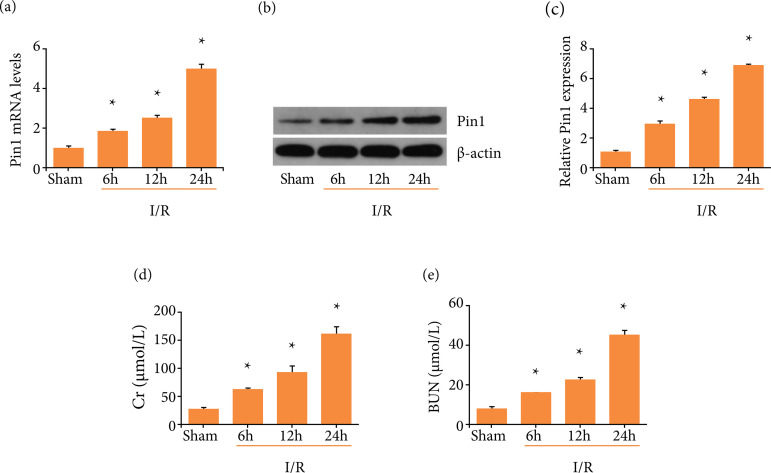
Pin1 expression was up-regulated in the kidney subjected to I/R in rats. **(a)** Pin1 mRNA level was detected by RT-PCR after ischemia 45 min and reperfusion 6, 12 and 24 h, respectively. **(b, c)** Pin1 protein level was detected by western blot and the quantification was performed. **(d, e)** Serum Cr and BUN level were detected after ischemia 45 min and reperfusion 6, 12 and 24 h, respectively. Values were expressed as the mean ± SEM (n = 8). *P < 0.05, relative to the Sham group.

RT-PCR results showed that I/R elevated Pin1 mRNA levels at 6, 12 and 24 h, with the highest expression at 24 h in comparison with the Sham group (Fig. 1a). Also, western blot showed a similar trend to RT-PCR, indicating that Pin1 levels up-regulated during the process of renal I/R (Fig. 1b and c). The renal function results indicated that experimental animals were susceptible to I/R injury, as demonstrated by the accumulated concentrations of Cr and BUN at I/R 24h (Fig. 1d and e). Overall, these data showed that Pin1 alterations might correlate with the I/R-injured kidney dysfunction.

### Pin1 inhibition protected kidney against I/R

**Figure 2:**
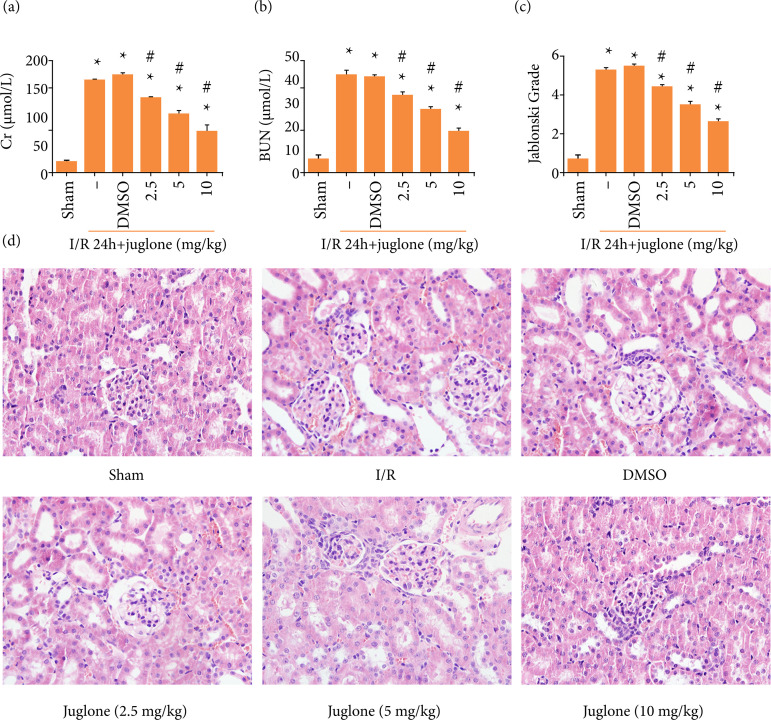
Pin1 inhibitor protected kidney against I/R injury in rats. **(a, b)** The effect of Juglone at different concentration (2.5, 5 and 10 mg·kg^–1^) on renal function in rats subjected to ischemia 45 min and reperfusion 24 h. **(c)** The effect of Juglone at different concentration (2.5, 5 and 10 mg·kg^–1^) on renal structure damage detected by H&E staining (400×). Values were expressed as the mean ± SEM (n = 8). *P < 0.05, relative to Sham group;

Treatment with different concentration (2.5, 5, 10 mg·kg^–1^) of juglone, a well-known Pin1 inhibitor, obviously prevented I/R-mediated Cr and BUN accumulation (Fig. 2a and b). Morphology changes were also shown. Acute tubular injury was induced by I/R stimuli, as evidenced by the obvious tubular expansion, tubular epithelium swell and collapse of the brush border. Treatment with Pin1 inhibitor prevented the tubular cells damages in a dose-dependent manner (Fig. 2c). Therefore, these results indicated that suppression of Pin1 conveyed protection to kidney suffered from I/R attack and we chose 10 mg·kg^–1^ as the experimental dose.

### Pin1 suppression attenuated Nrf2/HO-1 pathway and endoplasmic reticulum stress mediated by I/R

**Figure 3:**
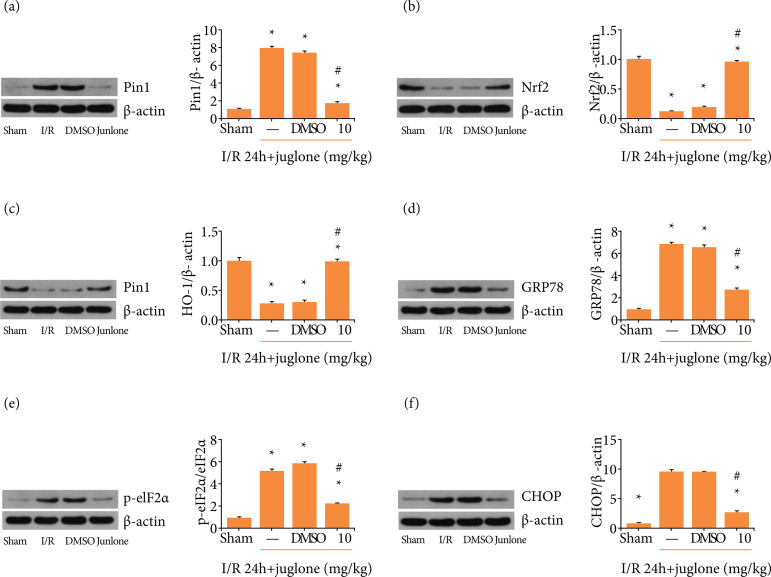
Pin1 inhibitor ameliorates endoplasmic reticulum stress and Nrf-2/HO1 pathway. **(a)** The effect of Pin1 inhibitor (10 mg·kg^–1^) on Pin1 expression in rats exposed to renal ischemia 45 min and reperfusion 24 h, and quantification was performed. **(b, c)** The effect of Pin1 inhibitor (10 mg·kg^–1^) on Nrf2-HO-1 pathway expression in rats exposed to renal ischemia 45 min and reperfusion 24 h, and quantification was performed. **(d–f)** The effect of Pin1 inhibitor (10 mg·kg^–1^) on GRP78, eIF2α and CHOP expression in rats exposed to renal ischemia 45 min and reperfusion 24 h, and quantification was performed. Values were expressed as the mean ± SEM (n = 8). *P < 0.05, relative to Sham group;

Western-blot demonstrated that Pin1 expression were inhibited by its inhibitor at the concentration of 10 mg·kg^–1^ (Fig. 3a). Then, the correlation of Pin1, Nrf2/HO-1 and ER stress was determined during I/R. As shown in the Fig. 3b and c, Pin1 inhibitor significantly rescued the down-regulation of Nrf2/HO-1 in the presence of I/R. In addition, ER stress-associated protein levels, including GRP78, p-eIF2α/ eIF2α and CHOP, were elevated in I/R-injured kidney, and juglone treatment prevented I/R-mediated up-regulated ER stress-associated protein levels (Fig. 3d–f). Taken together, Pin1 inhibition prevented Nrf2/HO-1 down-regulating and restricted ER stress in the context of I/R.

### Pin1 expression was elevated after H/R process in vitro

**Figure 4:**
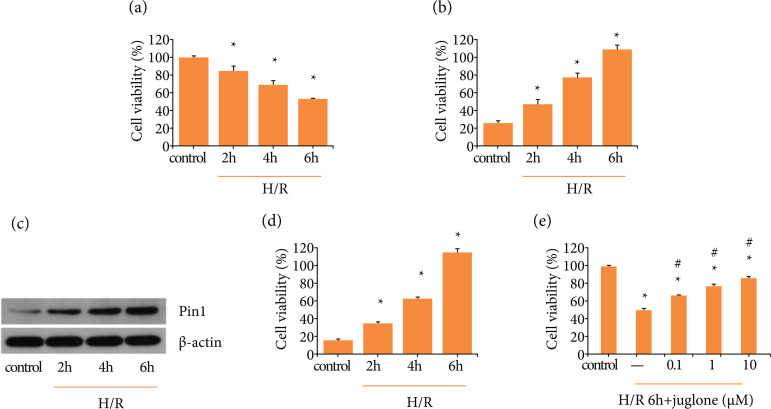
Pin1 expression was elevated during H/R process in HK-2 cells and Pin1 inhibitor could protected cell ability against H/R. **(a)** Cell viability was detected at hypoxia 12 h and reoxygenation 2, 4 and 6 h, respectively. **(b)** Pin1 mRNA level was detected by RT-PCR after hypoxia 12 h and reoxygenation 2, 4 and 6 h, respectively. (c, d) Pin1 protein level was detected by western blot and the quantification was performed. **(e)** Cell viability was detected with the treatment of different concentration of Pin1 inhibitor at hypoxia 12 h and reoxygenation 6 h. Values were expressed as the mean ± SEM (n = 8). *P < 0.05, relative to control group;

First, whether various reoxygenation time was correlate to cell viability was assessed using CCK8 assay. The cell viability at all reoxygenation time points, including 2, 4 and 6 h, was remarkably reduced by comparing to control, especially at reoxygenation 6 h (Fig. 4a). Both mRNA and protein levels of Pin1 were up-regulated in cells suffered from H/R at all time points (Fig. 4b–d). Therefore, we chose H/R 6 h in the following experiments. Besides, with the treatment of Pin1 inhibitor (0.1, 1 and 10 μmol·L^–1^) prior to H/R, it showed that Pin1 inhibition could protected cell viability that impaired by H/R (Fig. 4e).

### Inhibition of Pin1 restricted ER stress induced in vitro

**Figure 5:**
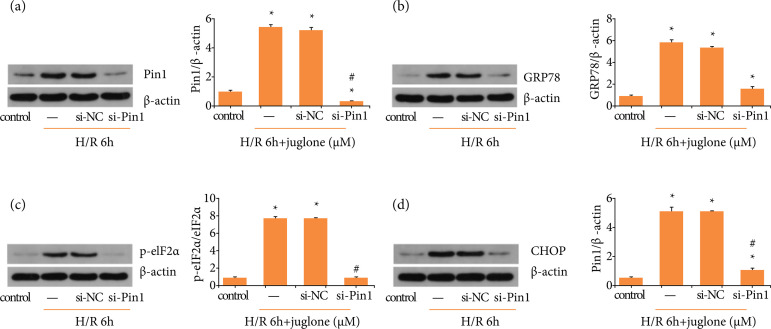
Pin1 silence alleviated endoplasmic reticulum stress induced by H/R. **(a)** HK-2 cells were transfected with negative control or si-RNA for Pin1 and subjected to H/R after transfection 24 h. Western blot was performed for Pin1 expression and quantification relative to control group. **(b–d)** The effect of si-Pin1 on the expression of GRP78, p-eIF2α, and CHOP after H/R and quantification relative to control group. Values were expressed as the mean ± SEM (n = 8). *P < 0.05, relative to control group;

Western-blot indicated that si-Pin1 suppressed Pin1 levels in H/R (Fig. 5a). Next, we found that H/R aggravate the expression of ER stress-related proteins and Pin1 knockdown significantly prevented ER stress-related proteins elevating in H/R stimuli (Fig. 5b–d).

### Pin1modulated ER stress dependent on Nrf2/HO-1.

**Figure 6:**
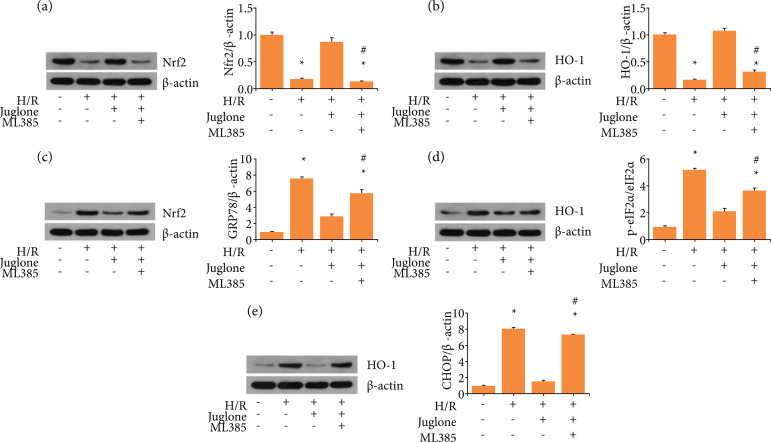
Pin1 regulates endoplasmic reticulum stress induced by H/R through Nrf2/HO-1 pathway. HK-2 cells were treated with Pin1 inhibitor (10 μmol·L^–1^) for 1 h, followed by H/R, with or without pretreatment with Nrf2 inhibitor ML385 (5 μmol·L^–1^). **(a, b)** The expression of Nrf2/HO-1 pathway was detected by western blot and quantification was performed. **(c–e)** The expression of GRP78, p-eIF2α, and CHOP were detected by western blot and quantification was performed relative to control group. Values were expressed as the mean ± SEM (n = 8). *P < 0.05, relative to control group;

Nrf2/HO-1 expression was reduced after H/R (Fig. 6a and b), which was partially reversed by Pin1 inhibitor, juglone, at the concentration of 10 μmol·L^–1^. ML385, Nrf2 inhibitor, was applied to further clarify the correlation between Pin1 and ER stress. Combined treatment with juglone and ML385 limited the elevation of Nrf2/HO-1 compared with juglone treatment only. Besides, it indicated that juglone restricted the up-regulation of ER stress-related protein levels by H/R. However, the combined treatment with juglone and ML385 significantly reduced the ER stress-related protein levels (Fig. 6c–e). Therefore, these findings indicated that Pin1modulated ER stress partially through Nrf2/HO-1.

## Discussion

We emphasized our work on the role of Pin1 in renal I/R, and explored the related mechanism. The results showed that Pin1 might be implicated in I/R-injured kidney. Simultaneously, inhibition of Pin1 afforded nephroprotection against I/R-impaired kidney function and tissue damage. Besides, Pin1 levels were elevated after H/R in vitro, and inhibition of Pin1 prevented endoplasmic reticulum stress induced by H/R. Moreover, further study demonstrated that endoplasmic reticulum stress was regulated by Pin1 independent on Nrf2/HO-1 pathway. Therefore, these novel findings showed that Pin1 might be a promising therapeutic target for renal I/R.

Pin1 has been considered as a key factor for regulating biological processes through modifying the targeted protein structure. It could bind to a p-Ser/Thr-Pro motif, catalyze cis-trans isomerization and also add another level of posttranslational regulation^13^^,^ 14. Previous study showed that the elevated expression of Pin1 promotes the pathogenesis and progression of cerebral and intestinal I/R injury^6^. Our study showed the consistent results with the previous reports.

Also, we investigated the different concentrations of Pin1 inhibitor against I/R and H/R injury. Inhibition of Pin1 could alleviate impaired renal function and structure damage induced by I/R. The in vitro experiments also showed that Pin1 inhibitor could protect the impaired HK-2 cell viability induced by H/R. Therefore, inhibition of Pin1 could protect against renal I/R injury. The negative control (no intervention or no nephrectomy) was not grouped in this study. Based on our previous research, it had no differences on renal function or structural damage between sham-operated group and negative control group.

Ischemia-reperfusion is the serious complication that usually happen in kidney transplantation, partial nephrectomy, heart surgeries and other major operations^6^. The relevant mechanisms of renal I/R include necrosis, mitochondrial dysfunction, apoptosis, ER stress, and oxidative stress^15^. Among them, ER stress is considered as one of the key mechanisms. Previous study reported that dexmedetomidine treatment alleviated myocardial infarction-mediated AKI through restricting endoplasmic reticulum stress^16^. Another study showed that renal I/R-impaired injury was prevented by fibroblast growth factor2 treatment dependent on the modulation of endoplasmic reticulum stress by activation of PI3K/AKTand MEK-ERK1/2 signals^17^. Our study demonstrated that the expression of ER stress-associated proteins was elevated after I/R and H/R process. Furthermore, the inhibition of Pin1, with inhibitor in vivo or si-RNA in vitro, could alleviate the elevated expression of ER stress-associated proteins induced by I/R or H/R. These results illustrated that Pin1 aggravated renal I/R injury through upregulation of ER stress in vivo and in vitro.

Under the physiological condition, Nrf2 was located and combined with Keap1 protein in the cytoplasm. When under various stresses stimulation, Nrf2 was detached from Keap1 and translocated into the nucleus, and then promoted HO-1 expression^18^. Previous studies discovered that Nrf2/HO-1 pathway was associated with ER stress during I/R process. It was reported that the elevated Nrf2/HO-1 reduced I/R-induced cardiac cell apoptosis and myocardial injury through alleviating endoplasmic reticulum stress-related signal molecules^19^. Another study showed that helix B position peptide played a key role in reducing acute lung injury by activating Nrf2/HO-1, which restricted ER stress in lung epithelial cells^20^. We showed that Nrf2/HO-1 levels were down-regulated and ER stress-related proteins were elevated upon I/R stimuli and inhibition of Pin1 activated Nrf2/HO-1 signals, thus protecting kidney against I/R stimulation. To further demonstrate the relationship between Nrf2/HO-1 and ER stress, we performed the in vitro experiments with Pin1 inhibitor and Nrf2 inhibitor. The combination treatment with Pin1 inhibitor and Nrf2 inhibitor could reverse the decreased endoplasmic reticulum stress-related protein expression induced by Pin1 inhibition, which indicated that Nrf2/HO-1 played a key role in the regulation between Pin1 and endoplasmic reticulum stress.

## Conclusion

Inhibition of Pin1 afforded protection against I/R injury and restricted renal injury dependent on the regulation of Nrf2/HO-1. This effect might occur through modulation of ER stress upon I/R stimuli. These results indicated that Pin1 was promising therapeutic target for renal I/R injury.
